# Simultaneous estimation of absolute concentrations of chromophores and the differential pathlength factor in forearm muscle using spectral derivatives

**DOI:** 10.1364/BOE.585939

**Published:** 2026-01-23

**Authors:** Jeremy C. Hebden

**Affiliations:** Department of Medical Physics & Biomedical Engineering, University College London, London, WC1E 6BT, UK

## Abstract

A method for estimating the absolute concentrations of chromophores in highly scattering tissues using near-infrared spectra is presented, which also yields estimates of the differential pathlength factor (DPF) and the power-dependency of scattering on wavelength. It involves measuring the attenuation spectral gradient and comparing it with an expression derived from diffusion theory. The validity of the approach is first explored using a diffusion model of light propagation in a homogenous slab, which is used to simulate measurements of diffuse reflectance in the adult forearm muscle during a vascular occlusion. Thereafter, the method is applied to experimental measurements performed on the forearms of five volunteers. The simulation results suggest that accuracy is significantly enhanced if some derived parameters are constrained to ranges which are physiologically realistic, and that the absolute concentrations of oxy- and deoxy-hemoglobin are estimated to within 40% and 20% of the true values, respectively. Furthermore, the wavelength-averaged DPF can be estimated to within around 10%. The measurements on volunteers revealed broadly consistent concentrations of the hemoglobins in the range 2-105 µM, and differential pathlength factors in the range 2.2-5.1.

## 1. Introduction

Since first proposed by Jöbsis in 1977 [1], near-infrared spectroscopy (NIRS) has been widely used to measure changes in the concentrations of oxy- and deoxy-hemoglobin (HbO_2_ and Hb) non-invasively, and thus provide a valuable indictor of tissue oxygenation. NIRS exploits the significant differences between the characteristic absorptions of these and other chromophores at near-infrared (NIR) wavelengths. A typical measurement involves coupling a broadband source and a detector to the tissue surface via optical fibers or fiber bundles, and analyzing the received light that has been scattered within the underlying tissues. However, measurements of the diffusely reflected light are dependent on the degree of scattering as well as absorption, and are also highly sensitive to the uncertain and variable coupling of light into and out of the tissue surface. These factors inhibit the routine derivation of absolute concentrations of the chromophores, and consequently most NIRS technology relies on measuring changes in signal under conditions where it can be assumed that the scattering and coupling have remained constant. The changes in signal can then be used to estimate changes in chromophore concentrations.

While methods have been evaluated to identify and/or eliminate artefacts in recorded data due to variable coupling [2,3,4,5], alternative types of optical measurement have been explored which are potentially less sensitive to coupling variability. Specifically, Dehghani *et al* [6,7]. and Pucci *et al* [8]. have noted the insensitivity of spectral derivatives to coupling. More recently, a method known as wavelength-modulated NIRS has been proposed which measures the spectral derivative directly [9], for example by modulating the source wavelength at a fixed frequency. Variation in tissue absorption over the wavelength range will produce a small modulation in detected intensity which can be measured using lock-in or Fourier methods.

The objective of the work described here has been to examine the potential of measurements of spectral derivatives to yield absolute concentrations of chromophores in (assumed homogeneous) highly scattering tissues. Section 2 presents the underlying theory, which suggests that, by employing a commonly-applied power-law model of the wavelength dependence of scattering, spectral derivatives may also yield the exponent of the model and the so-called differential pathlength factor (DPF) which features in NIRS calculations, and which hitherto it has been necessary to estimate or measure independently. In section 3, the approach is explored using a model of light propagation in a homogenous slab based on analytical solutions to the time-dependent diffusion equation. This model is employed to simulate measurements of diffuse reflectance in the adult forearm muscle during a vascular occlusion. Thereafter, the method is applied to experimental measurements performed on the forearms of healthy volunteers during a vascular occlusion, which are described in section 4.

## 2. Theory

To acquire a typical NIRS measurement, two optical fibers are placed in contact with the surface of the interrogated tissue, separated by a distance *d*, where one fiber delivers light to the surface, while the other conveys received light to a detector. For a homogeneous tissue, the detected intensity *I* is commonly described by the modified Beer-Lambert law [10,11] as follows:

(1) $$I=k\;I_{0}\;\exp(-\mu_{a}\beta d-G)$$

where µ_a_ is the tissue absorption coefficient, *β* is called the differential pathlength factor (DPF), *G* is an unknown scatter-dependent geometric factor, *I_0_* is the source intensity, and *k* is the (unknown and highly variable) coupling efficiency. The DPF is equal to the average pathlength of detected photons divided by *d*, and the factor exp(*–G*) describes the reduction in detected intensity due to scatter. The value of µ_a_ depends on the concentrations *c_n_* of the chromophores as follows:

(2) $$\mu_{a}=\sum_{n}\varepsilon_{n}c_{n}+\mu_{b}$$

where *n* is the number of chromophores, *ε_n_* are their molar extinction coefficients, and µ_b_ describes any residual background absorption which has no (or unknown and therefore assumed no) wavelength dependence. Because *k* and *G* are unknown, the applications of NIRS are mostly restricted to observing changes in chromophore concentration and oxygenation, under conditions where it is assumed that *k* and *G* remain constant. The attenuation *A* of light between the source and detector can be expressed as:

(3) $$A=-\ln\left(\frac{I}{I_{0}}\right)=\mu_{a}\beta d+G-\text{ln}(k)$$

Differentiating Eq. (3) with respect to wavelength *λ* yields:

(4) $$\frac{\partial A}{\partial \lambda }=\frac{1}{I_{0}}\frac{\partial I_{0}}{\partial \lambda }-\frac{1}{I}\frac{\partial I}{\partial \lambda }=d\left(\beta \frac{\partial \mu_{a}}{\partial \lambda }+\frac{\partial \beta }{\partial \lambda }\mu_{a}\right)+\frac{\partial G}{\partial \lambda }-\frac{1}{k}\frac{\partial k}{\partial \lambda }$$

It is evident from this equation that the rate of change of attenuation *A* with wavelength *∂A*/*∂λ* is independent of the absolute magnitude of the measured intensity *I*. Thus, assuming that the unknown coupling has no wavelength dependency (i.e. *∂k*/*∂λ* = 0), a measurement of *∂A*/*∂λ* should be independent of the coupling. Experiments on highly scattering samples and models of light transport in turbid media [12] have demonstrated that measurements of attenuation spectral gradient can be employed to estimate absolute values of absorbing properties using a prior estimate of the DPF. These studies empirically observed that, for homogenous media, Eq. (4) can be simplified as:

(5) $$\frac{\partial A}{\partial \lambda }\approx d<\beta >\sum_{n}\frac{\partial \varepsilon_{n}}{\partial \lambda }c_{n}-\alpha \lambda^{-p}$$

where *α* and *p* are positive constants and <β> is the wavelength-averaged value of the DPF. The term on the right-hand side accommodates the wavelength dependence of the transport scatter coefficient µ_s_′, where empirical models published elsewhere [13,14] indicate a relationship of the form µ_s_′(*λ*) = Q*λ*^–p^.

Equation (5) was found to be highly robust to changes in measurement geometry providing that such changes are reflected in the estimate of the mean DPF (i.e. no independent assessment of the parameter *G* is required). Given that *d*, <β> , and *∂ε_n_*/*∂λ* are either known or can be reliably estimated, measurements of *∂A*/*∂λ* at multiple discrete wavelengths can be used to derive the concentrations *c_n_* and the constants *α* and *p* by solving Eq. (5) as a matrix equation or using a minimum norm least-squares approach. Experimental and modelling results suggested that chromophore concentrations in homogeneous tissue-like turbid media could be estimated with a typical accuracy of better than 10% [12], comparable to the accuracy achieved by Dehghani *et al* [6]. using spectral derivatives estimated from simulated tissue data. Equation (5) has also been applied to data recorded on a tissue-equivalent phantom containing a series of embedded targets with different concentrations of absorbing dye, and coated with a layer of dark hair [15]. The phantom was imaged using a broadband source and spectrometer, scanned over the surface, and prior knowledge of the absorbing characteristics of the dye and of melanin (the dominant absorber in hair) were used to reveal the targets with remarkable clarity.

An expression for the attenuation spectral gradient *∂A*/*∂λ* can also be derived from the analytical model presented by Contini *et al* [16]., based on solutions to the time-dependent diffusion equation for a semi-infinite homogeneous medium. Using Eqs. (51) and (52) of [16], the diffuse reflectance may be expressed as:

(6) $$R(d)\approx \frac{z_{e}+z_{0}}{2\pi d^{3}}.\left(1+d\sqrt{\frac{\mu_{a}}{D}}\right).exp\left(-d\sqrt{\frac{\mu_{a}}{D}}\right),$$

and the DPF expressed as:

(7) $$\beta \approx \frac{d}{2D}.{\left(1+d\sqrt{\frac{\mu_{a}}{D}}\right)}^{-1},$$

where *d* is the source-detector separation on the surface, *z_0_* = 1/µ_s_′, *z_e_* = 2*A*(*n_r_*)/3µ_s_′, D = 1/3µ_s_′, and *A*(*n_r_*) depends on the relative refractive index *n_r_* at the boundary. The above formulae assume that *d* >> (2*z_e_* + *z_0_*), or that:

(8) $$d\gg \frac{1}{\mu_{s}^{\prime }}\left(\frac{4}{3}A(n_{r})+1\right).$$

Using Eq. (A3) of [16], *A*(1.4) ≈ 3.0. Thus for a tissue-like value µ_s_′ ≈ 1 mm^−1^ this implies that *d* >> 5 mm. By differentiating *R*(*d*) with respect to wavelength, it is found that the attenuation gradient can be expressed as:

(9) $$\frac{\partial A}{\partial \lambda }=-\frac{\partial }{\partial \lambda }\ln R=-\frac{\partial R}{\partial \lambda }/R\approx d\beta \frac{\partial \mu_{a}}{\partial \lambda }+\frac{1}{\mu_{s}^{\prime }}\frac{\partial \mu_{s}^{\prime }}{\partial \lambda }(1+d\beta \mu_{a}).$$

Using a scattering model µ_s_′ = Q*λ*^–*p*^ implies that (*∂*µ_s_′/*∂λ*)/µ_s_′ = –*p*/*λ*. Thus:

(10) $$\frac{\partial A}{\partial \lambda }\approx d\beta \frac{\partial \mu_{a}}{\partial \lambda }-\frac{p}{\lambda }(1+d\beta \mu_{a}).$$

This result has similarities with the empirically-derived result as given in Eq. (5), including the observation that the right-hand terms disappear when scatter has no wavelength dependence (i.e. when *p* = 0), and their wavelength dependences are the same when *p* ≈ 1. Inserting Eq. (2) and $\frac{\partial \mu_{a}}{\partial \lambda }=\sum_{n}\frac{\partial \varepsilon_{n}}{\partial \lambda }c_{n}$ into Eq. (10) yields:

(11) $$\frac{\partial A}{\partial \lambda }\approx d\beta \sum_{n}c_{n}\left(\frac{\partial \varepsilon_{n}}{\partial \lambda }-\frac{p}{\lambda }\varepsilon_{n}\right)-\frac{p}{\lambda }(1+d\beta \mu_{b}).$$

The analysis up to this point assumes that the DPF is a function of wavelength *β* (λ). However, given that experimental evidence [17] suggests the variation in DPF with wavelength is relatively small (compared with the wavelength dependence of absorption), henceforth *β* in Eq. (11) will be substituted with its wavelength averaged value < *β* > as follows:

(12) $$\frac{\partial A}{\partial \lambda }\approx d<\beta >\sum_{n}c_{n}\left(\frac{\partial \varepsilon_{n}}{\partial \lambda }-\frac{p}{\lambda }\varepsilon_{n}\right)-\frac{p}{\lambda }\left(1+d<\beta >\mu_{b}\right).$$

Thus it is hypothesized that, assuming prior knowledge of the specific absorption coefficients and their spectral gradients, measurements of the attenuation gradient at a minimum of *n* + 3 discrete wavelengths is sufficient in principle to solve for *n* chromophore concentrations, the scattering power *p*, the constant absorption parameter µ_b_, and the mean DPF. In practice, however, to ensure a unique solution, it is important that those discrete wavelengths should correspond to spectral regions where the coefficients and their gradients are distinct from each other.

In the following section, an attempt to test this hypothesis is described using the same diffusion-based model (although based on a slightly different set of equations) used to generate Eq. (11). The specific objective is to explore the extent to which the unknown coefficients and other parameters can be derived independently from the attenuation spectral gradient *∂A*/*∂λ*, and investigate when and to what degree prior knowledge of one or more parameters is required to estimate the others.

## 3. Validation using an analytical model of photon migration for a turbid slab

### 3.1. Model description

**Fig. 1.**
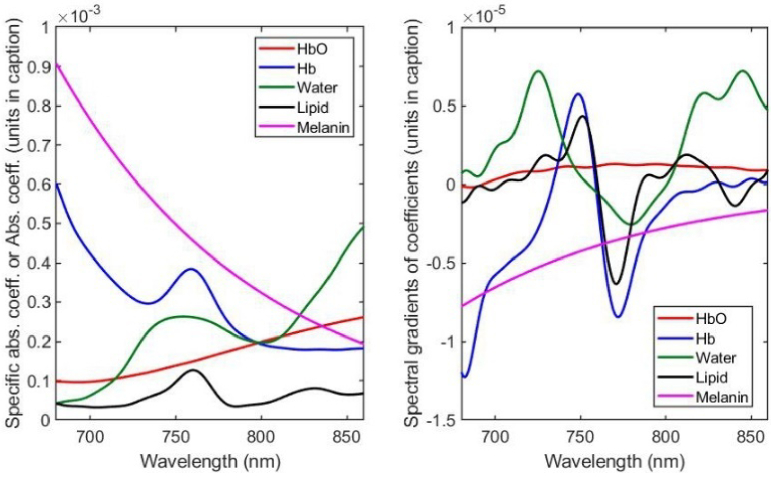
a) Specific absorption coefficients of HbO_2_ (mm^−1^M^−1^), Hb (mm^−1^M^−1^), and melanin (mm^−1^M^−1^ / 100); the absorption coefficients of water (mm^−1^ / 10) and lipid (mm^−1^ / 10). b) Spectral gradients of the specific absorption coefficients HbO_2_ (mm^−1^M^−1^nm^−1^), Hb (mm^−1^M^−1^nm^−1^), and melanin (mm^−1^M^−1^nm^−1^ / 100); the spectral gradients of the absorption coefficients of water (mm^−1^nm^−1^ / 10) and lipid (mm^−1^nm^−1^ / 10).

The analysis of Contini *et al* [16]. mentioned above provides a set of solutions to the time-dependent diffusion equation for a variety of measurement geometries. Whereas Eqs. (6) and (7) derive from a semi-infinite homogeneous medium, an initial attempt at validating the approximation in Eq. (12) has been attempted using solutions for a slab geometry of finite thickness. Specifically, the wavelength-dependent attenuation *A*(*λ*) and differential pathlength factor *β* (*λ*) were estimated for a source and detector separated by a distance *d* = 11 mm on the same surface of a slab of thickness 50 mm, with lateral dimensions extending to infinity. The internal and external refractive indices of the slab were 1.4 and 1.0 respectively. The wavelength-dependent absorption coefficient of the slab was given by:

(13) $$\mu_{a}=\epsilon_{HbO_{2}}c_{HbO_{2}}+\epsilon_{Hb}c_{Hb}+\epsilon_{Mel}c_{Mel}+\mu_{a,Water}W+\mu_{a,Lipid}L+\mu_{b},$$

where *ε_HbO2_* and *c_HbO2_* are the specific absorption coefficient and molar concentration of oxyhemoglobin, *ε_Hb_* and *c_Hb_* are the equivalent parameters for deoxyhemoglobin, and *ε_Mel_* and *c_Mel_* are those for melanin. The parameters µ_a,Water_ and µ_a,Lipid_ are the absorption coefficients of water and lipid, and *W* and *L* are the fractions of water and lipid. Estimates of the three extinction coefficients and two absorption coefficients as a function of wavelength (in the range 680 nm – 860 nm) are displayed in Fig. 1(a) using data extracted from the online database compiled by Prof. Scott Prahl of the Oregon Institute of Technology [18]. The extinction coefficient of melanin has been modelled here by assuming an exponential fit to the values cited by Sarna and Swartz [19]. Note that while melanin concentrations in tissue are relatively small, prior studies [15] have already shown that its wavelength dependence cannot be ignored for measurements which may involve transmission through hair. To aid visual comparison, some displayed coefficients are divided by appropriate factors of ten, as indicated in the caption. Shown in Fig. 1(b) are the first derivatives of these spectra with respect to wavelength, obtained by fitting a 32^nd^-order polynomial to each spectrum, and then finding the gradient of the polynomial.

The transport scatter coefficient was modelled using µ_s_′ = 1.0 (*λ*/800)^−*p*^ mm^−1^, where *p* is positive number and wavelength *λ* is expressed in nanometers, based on an empirically observed wavelength dependence [13,14].

**Fig. 2.**
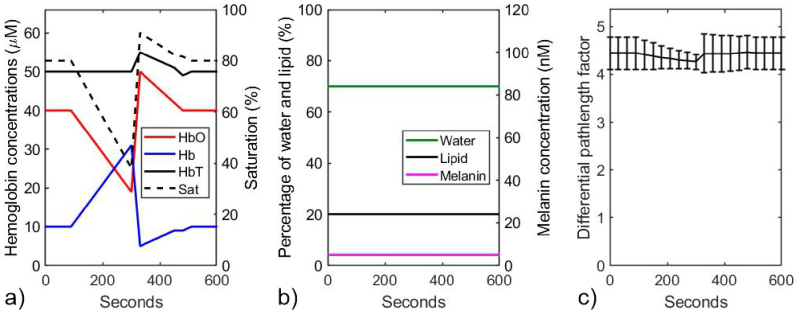
a) Modelled variation in concentrations of HbO_2_, Hb, HbT, and SO_2_ during a vascular occlusion. b) The constant percentages of water and lipid during the vascular occlusion and the constant melanin concentration. c) The derived mean DPF. The error bars indicate the range of values due to the variation in wavelength.

The model was used to represent an ideal homogeneous forearm muscle during a vascular occlusion. There are few reliable published measurements of average hemoglobin concentration in the forearm, but based on typical adult hemoglobin concentrations in whole blood and rough estimates of average blood concentration in muscle, a steady state total hemoglobin (HbT) concentration of 50 µM was chosen, and a steady state oxygen saturation (SO2, the fraction of HbO_2_ relative to HbT) of 80%. Following trends observed in several published estimates of the changes in concentrations of Hb, HbO_2_, and HbT during vascular occlusion [20,21,22], these parameters were modelled to vary over a period of 600 seconds as illustrated in Fig. 2(a). The occlusion is applied after 90 seconds, and removed after 300 seconds. Meanwhile, the percentage of water and lipid are held constant at 70% and 20% respectively, and the melanin concentration (assumed uniformly distributed) is held at a somewhat arbitrary 5 nM. This is illustrated in Fig. 2(b).

Using these data, and a wavelength-independent absorption coefficient µ_b_ = 0.012 mm^−1^, Eq. (13) was used to derive values of the absorption coefficient of the slab model at time intervals of 30 seconds and at 1 nm wavelength intervals between 680 nm and 860 nm. Then, using a constant scattering power *p* = 1.2, Eq. (7) was used to derive the DPF at the same time points and at each wavelength. The mean DPF at each time point is displayed in Fig. 2(c), where the error bars indicate the range of values due to the variation in wavelength. The relatively small percentage variation in DPF with wavelength tends to support the approximation implicit in Eq. (12) that it can be substituted with its wavelength averaged value < *β* >.

**Fig. 3.**
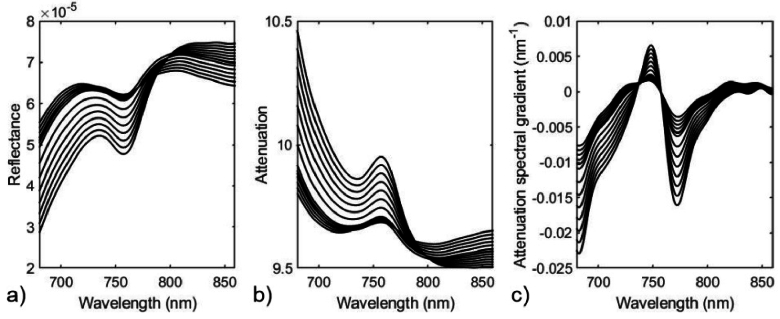
a) Diffuse reflectance spectra calculated for the slab model at 30 second time intervals using the model data shown in Fig. 1(a) and Fig. 2 (arbitrary units). b) Reflectance spectra converted to values of attenuation. c) The spectral gradients of the attenuation.

Next, Eq. (6) was used to derive the wavelength dependence of the diffuse reflectance *R*(*λ*), acquired at a distance *d* = 11 mm from the source, for each time point. The reflectance spectra for all 21 time points is shown in Fig. 3(a). These spectra were converted to attenuation, using *A*(*λ*) = –ln[*R*(*λ*)], as illustrated in Fig. 3(b), and then the attenuation spectral gradient *∂A*/*∂λ* was computed using a 32^nd^-order polynomial fit to the values of *A*(*λ*), as shown in Fig. 3(c). Note that the units of reflectance are arbitrary, and do not influence the attenuation gradient.

### 3.2. Results

The objective of this modelling exercise is to determine to what extent the chromophore concentrations, and other unknown parameters, can be estimated from the attenuation gradients shown in Fig. 3(c) by expressing Eq. (12) as a matrix equation and applying a nonlinear least-squares solver. In all cases, the known source-detector separation *d* = 11 mm was used. The results described below are obtained under three different conditions.

#### 3.2.1. All parameters are variable and unconstrained

**Fig. 4.**
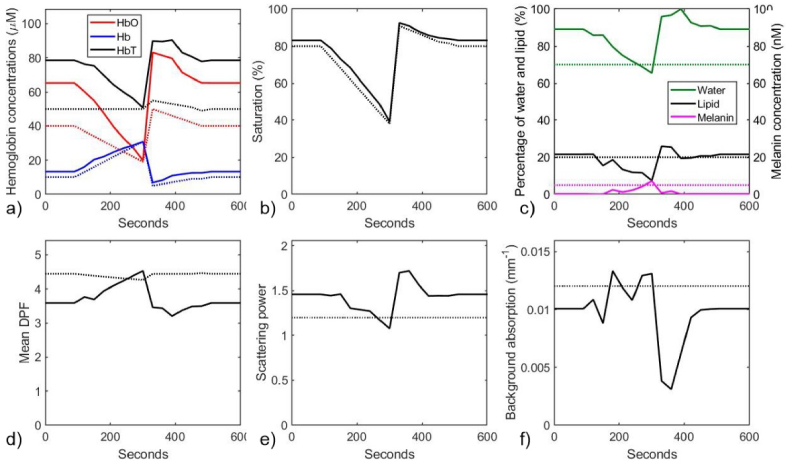
Model parameters estimated at 30-second time points during the simulated vascular occlusion, using a nonlinear least squares fit where all parameters are allowed to vary. a) Estimated (solid lines) and true (dotted lines) concentrations of HbO_2_, Hb, and HbT; b) Estimated (solid line) and true (dotted line) values of SO_2_. c) Estimated (solid lines) and true (dotted lines) percentages of water and lipid, and of melanin concentration. d) Estimated (solid line) and true (dotted line) values of the mean DPF. e) Estimated (solid line) and true (dotted line) values of the scattering power *p*. f) Estimated (solid line) and true (dotted line) values of the wavelength-independent absorption coefficient µ_b_.

Using the coefficients and their gradients shown in Fig. 1, a nonlinear least squares fit of Eq. (12) to the model data in Fig. 3(c) was performed over the wavelength range 680 nm – 860 nm to obtain estimates of all the model parameters at each 30-second time point during the simulated vascular occlusion. These estimates are displayed in Fig. 4, with the true values indicated by the dotted lines.

It is evident that without any constraints being applied, significant variation is observed in all the parameters during the simulated occlusion, whereas only the hemoglobin concentrations, the saturation, and the average DPF vary when generating the simulated data as shown in Fig. 3. Although the concentrations of HbO_2_ and Hb exhibit the expected trends, their absolute values differ from the true values by up to 60%, and there is a strong artefactual decrease in HbT which coincides with similarly erroneous changes in other parameters.

#### 3.2.2. Maximum prior information: W, L, c_Mel_, p, and µ_b_ fixed at true values

**Fig. 5.**
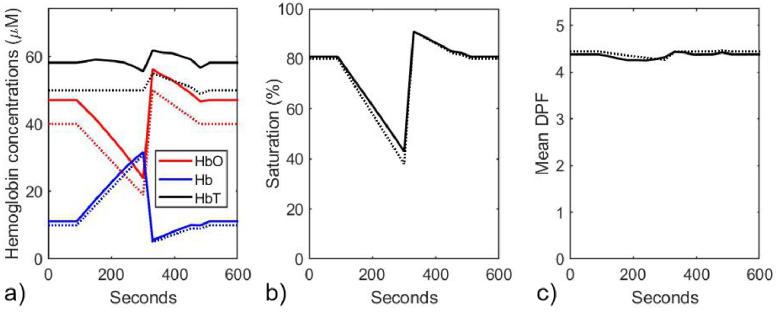
Model parameters estimated at 30-second time intervals during the simulated vascular occlusion, using a nonlinear least squares fit where the water and lipid fractions, the melanin concentration, the scattering power and the wavelength-independent absorption coefficient are fixed at their true values. a) Estimated (solid lines) and true (dotted lines) concentrations of HbO_2_, Hb, and HbT. b) Estimated (solid line) and true (dotted line) values of SO_2_. c) Estimated (solid line) and true (dotted line) values of the mean DPF.

A second nonlinear least squares fit of Eq. (12) to the model data in Fig. 3(c) was performed, but with the water and lipid fractions (*W* and *L*), the melanin concentration (*c*_Mel_), the scattering power (*p*) and the wavelength-independent absorption coefficient (µ_b_) held at their true values. The derived values of the hemoglobin concentrations, saturation, and the average DPF during the simulated vascular occlusion are displayed in Fig. 5 (solid lines), where they are compared with the true values (dotted lines).

As anticipated, the application of the constraints on the other five parameters have yielded more accurate estimates of the absolute concentration of the hemoglobins, and of the mean DPF during the simulated vascular occlusion. The values are not perfectly recovered, due to implicit approximations used to derive Eq. (11) and the assumption that the DPF in Eq. (11) can be replaced with a wavelength averaged value. The large artefactual decrease in HbT evident in Fig. 3(a) has largely disappeared, and differences between the derived and true absolute concentrations of the hemoglobins are between 2% and 25% of the true values.

#### 3.2.3. Limited prior information: application of physiologically-realistic constraints

Although precise knowledge of the parameters held at fixed values in section 3.2.2 will not be available, for most practical applications of NIRS some assumptions can reasonably be made about the concentrations of some chromophores and tissue scattering properties, and whether or not they vary over a period of time. For a vascular occlusion, it is usually considered reasonable to assume that the water, lipid, and melanin concentrations remain constant, and that the scattering properties do not change. Therefore this was used to constrain the nonlinear least squares fitting procedure in two stages as follows.

First, fits were performed with the water and lipid fractions *W* and *L*, and the scattering parameter *p*, constrained within ranges consistent with published estimates for the adult forearm. Unfortunately there is not a great abundance of such data, and values are often inconsistent. A particularly valuable resource is a paper by Nishimura *et al* [23]. which reports an estimate of about 52% water content in adult forearm muscle using both MRI and optical measurements. More generally, the water content in muscle is quoted at about 76% by Serra-Prat *et al* [24]. Lipid content is expected to be strongly variable between individuals, and study of human forearms using x-ray computed tomography by Maughan *et al* [25]. determined a lipid fraction of 15 ± 6% in males and 29 ± 6% in females. The parameter *p*, which describes the assumed power relationship between scattering and wavelength, has been explored experimentally, with Saager *et al* [13]. suggesting values of 1.2 ± 0.23 for healthy tissues. Meanwhile Taroni *et al* [26]. used spectroscopic techniques to determine this parameter in a variety of tissue types including the forearm, with values in the range 0.53–1.79. Based on the limited data available, the selected parameters ranges were *W* = 0.5-0.8, *L* = 0.15-0.3, and *p* = 0.7-1.8, which are consistent with the simulated values of *W* = 0.7, *L* = 0.2, and *p* = 1.2.

**Fig. 6.**
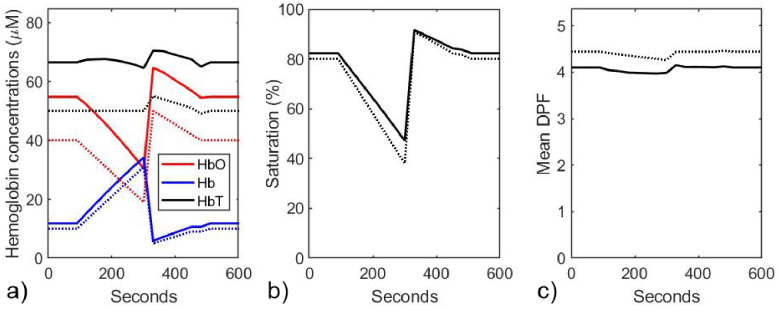
Model parameters estimated at 30-second time points during the simulated vascular occlusion, using a nonlinear least squares fit where the parameters *W*, *L*, *p*, *c_Mel_*, and µ_b_ are held constant at values from a prior fit where *W*, *L*, and *p* are constrained within physiologically-realistic ranges. a) Estimated (solid lines) and true (dotted lines) of concentrations of HbO_2_, Hb, and HbT. b) Estimated (solid line) and true (dotted line) value of SO_2_. c) Estimated (solid line) and true (dotted line) values of the mean DPF.

Second, the average values of these constrained parameters and of the unconstrained melanin concentration (*c*_Mel_) and the background absorption (µ_b_) over the full duration were calculated (yielding *W* = 0.78, *L* = 0.18, *p* = 1.354, *c_Mel_* = 2.1 nM, and µ_b_ = 0.0105 mm^−1^) and then the fitting process was then repeated while keeping these five parameters fixed at the average values. The resulting variations in the hemoglobin concentrations, saturation and average DPF are shown in Fig. 6.

The observed trends follow the true values very closely. The differences between the estimated and true values of the saturation and DPF are mostly within 10% percent, and the difference for Hb concentration is less than 20%. However, the estimated concentration of HbO_2_ is consistently around 40% higher than the true values. Given the absence of any other simple, non-invasive method of determining absolute hemoglobin concentrations in tissue, errors of up to 40% may be considered diagnostically useful in some clinical circumstances.

#### 3.2.4. Additional prior information: ΔHbT constrained to true values

As mentioned above, conventional NIRS methods are commonly used to derive changes in chromophore concentrations Δ*c_n_* from changes in attenuation Δ*A* assuming the coupling and scattering losses remain constant. This can be expressed as:

(14) $$\Delta A=\Delta \mu_{a}\beta d=\beta d\sum_{n}\varepsilon_{n}\Delta c_{n}.$$

**Fig. 7.**
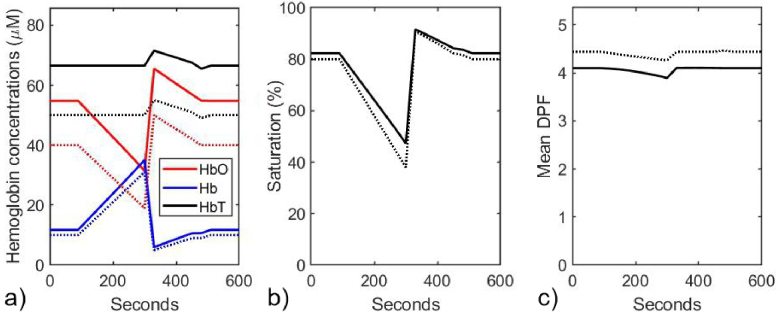
Model parameters estimated at 30-second time points during the simulated vascular occlusion, using a nonlinear least squares fit where the parameters *W*, *L*, *p*, *c_Mel_*, and µ_b_ are held constant at values from a prior fit where *W*, *L*, and *p* are constrained within physiologically-realistic ranges, and where changes in concentrations of HbO_2_ and Hb are constrained relative to time zero so that their sum is equal to the true changes in HbT. a) Estimated (solid lines) and true (dotted lines) of concentrations of HbO_2_, Hb, and HbT. b) Estimated (solid line) and true (dotted line) value of SO_2_. c) Estimated (solid line) and true (dotted line) values of the mean DPF.

This facilitates an additional source of prior information which might yield better estimates of the absolute concentrations using Eq. (12). This can be achieved by first using Eq. (14) to derive the changes in total hemoglobin concentration ΔHbT relative to an arbitrary time point before the start of the vascular occlusion, and then restricting the changes in the absolute concentrations of Hb and HbO_2_ so that ΔHbT = ΔHb + ΔHbO_2_. This was implemented for the model data using the known values of ΔHbT and the same constraints on the other parameters used to generate the result in Fig. 6. The outcome is displayed in Fig. 7, where changes ΔHb and ΔHbO_2_ are derived relative to the concentrations at time zero.

The additional prior information has produced only marginal improvements in the accuracy of the absolute values of the derived parameters, although the trends are improved, especially that of the mean DPF. Note that, alternatively, constraints on the change in saturation could be applied instead.

## 4. Experiments on forearms of healthy volunteers during a vascular occlusion

### 4.1. In vivo measurements

**Fig. 8.**
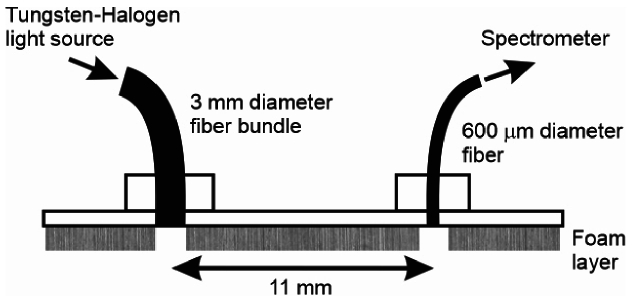
A probe used to acquire measurements of diffuse reflectance on the adult forearm. It supports a fiber bundle connected to a Tungsten-Halogen light source and a fiber coupled to the spectrometer, located 11 mm apart.

Sequences of diffuse reflectance spectra were recorded on the forearms of five light-skinned adult male volunteers during a vascular occlusion using an optical spectrometer and the simple probe illustrated in Fig. 8. The probe consists of a rigid plastic plate to which two optical fibers are secured, 11 mm apart. A 5 mm thick layer of light-absorbing foam is attached to the lower surface of the probe, with 5 mm diameter holes directly below each optical fiber. A spectrometer (Ocean Insight HR2000+ CG) was coupled to probe via a 600 µm diameter fiber, and a broadband light source (Ocean Insight Tungsten-Halogen HL-2000-HP) was coupled via a 3 mm diameter fiber bundle. The source delivers an output power of the order of 10 µW/nm at around 800 nm and the spectrometer has a spectral resolution of about 1 nm.

The probe was secured against the forearm using a band of elastic tape, approximately over the extensor digitorum muscle. Care was taken to avoid placement directly over obvious surface veins. A pneumatic cuff was wrapped around the upper arm. Spectra were then recorded every 30 seconds for about 4 minutes, after which the cuff was manually inflated to a pressure of 220 mmHg, and maintained at this pressure for about 5 minutes, during which further spectra were acquired at 30-second intervals. The cuff was then deflated, and spectra continued to be acquired every 30 seconds for an additional period of at least 4 minutes. The arm remained relaxed and stationary throughout. Meanwhile, a source spectrum was acquired separately by coupling the source directly to the spectrometer.

After subtraction of background noise, a 32^nd^−order polynomial was fitted to the logarithm of each spectrum between wavelengths of 650 nm and 900 nm, sufficient to account for subtle features in the broadband spectrum of the source. The gradient of each log spectrum at any wavelength within the fitted interval could then be estimated using the parameters of the corresponding polynomial. Values of the first derivative of attenuation with respect to wavelength (parameter *∂A*/*∂λ* in Eq. (12)) were then calculated by subtracting the gradient of the log of each spectrum from the corresponding gradient of the log of the source spectrum.

### 4.2. Data processing

Using the coefficients and their gradients shown in Fig. 1, a nonlinear least squares fit of Eq. (12) to the values of *∂A*/*∂λ* was performed over the wavelength range 680 nm – 860 nm to obtain estimates of all the parameters at each 30-second time point during the vascular occlusion. The simulation results described in section 3.2 showed that accommodating as much prior information as possible is highly advantageous, particularly to avoid spurious variation in optical parameters such as water and lipid concentrations. Therefore, for the processing of the experimental data, parameter estimation during each vascular occlusion involved three stages as follows.

a) First, nonlinear least squares fits were performed with the water and lipid fractions *W* and *L*, and the scattering parameter *p*, constrained within ranges consistent with published estimates for forearm muscle reported in section 3.2.3: *W* = 0.5-0.8, *L* = 0.15-0.3, and *p* = 0.7-1.8.
b) A second set nonlinear least squares fits were performed with the *W*, *L*, the melanin concentrations (*c*_Mel_), the scattering power (*p*), and the wavelength-independent absorption (µ_b_) all fixed at their average values obtained for the first set of fits in part (a). This effectively adds the constraint that none of these parameters change during the vascular occlusion.
c) Finally, following the process typically adopted for conventional NIRS, changes in attenuation Δ*A* (relative to time zero) were used to estimate the changes in the hemoglobin concentrations using Eq. (14) and mean DPF values derived in part (b). The processing in part (b) was then repeated using the derived changes in HbT to constrain the changes in concentrations of Hb and HbO_2_.

### 4.3. Results

**Fig. 9.**
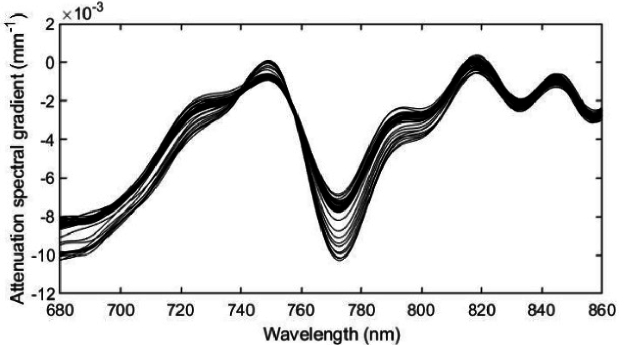
The spectral gradients of the attenuation measured at 30 second intervals on the forearm of an adult male volunteer during a vascular occlusion.

The outcomes of the data processing steps described above are illustrated here using a typical volunteer measurement (V1). Figure 9 shows the measurements of attenuation spectral gradient obtained from a set of 40 spectra acquired before, during and after the vascular occlusion. The data are in reasonable qualitative agreement with the model data shown in Fig. 3(c).

**Fig. 10.**
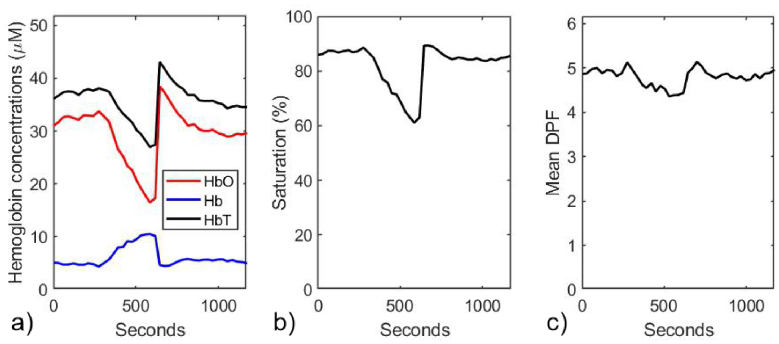
Parameters estimated at 30-second time points during the vascular occlusion on an adult volunteer, using a nonlinear least squares fit where the parameters *W*, *L*, *p*, *c_Mel_*, and µ_b_ are held constant at values from a prior fit where *W*, *L*, and *p* are constrained within physiologically-realistic ranges. a) Estimated concentrations of HbO_2_, Hb, and HbT. b) Estimated values of SO_2_. c) Estimated values of the mean DPF.

Using the coefficients and their gradients shown in Fig. 1, a nonlinear least squares fit of Eq. (12) to the data in Fig. 9 was performed over the wavelength range 680 nm – 860 nm to obtain estimates of all the model parameters at each 30-second time point when the water and lipid fractions and the scattering power were constrained to *W* = 0.5-0.8, *L* = 0.15-0.3, and *p* = 0.7-1.8 respectively. Next, the average values of these constrained parameters and of the melanin concentration and the background absorption over the full duration were calculated, yielding *W* = 0.52, *L* = 0.29, *p* = 1.27, *c_Mel_* = 22 nM, and µ_b_ = 0.0243 mm^−1^. Then the fitting process was repeated while keeping these five parameters fixed at the average values. The resulting variations in the hemoglobin concentrations, saturation and average DPF are shown in Fig. 10.

**Fig. 11.**
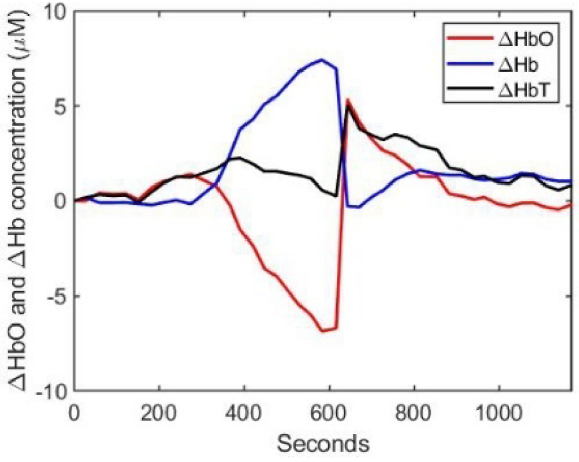
Estimated changes in HbO_2_, Hb, and HbT concentrations derived from changes in attenuation ΔA relative to time zero. This employed Eq. (14) and an assumption that the DPF was equal to the previously derived mean value shown in Fig. 10(c).

Noting that the total hemoglobin exhibits a marked (and probably artefactual) decrease during the occlusion, the measured spectra were reprocessed to estimate the changes in hemoglobin concentrations using Eq. (14) assuming all other factors remain constant, and the result is shown in Fig. 11. The DPF in Eq. (14) at each timepoint was assumed to be equal to the corresponding average value shown in Fig. 10(c).

**Fig. 12.**
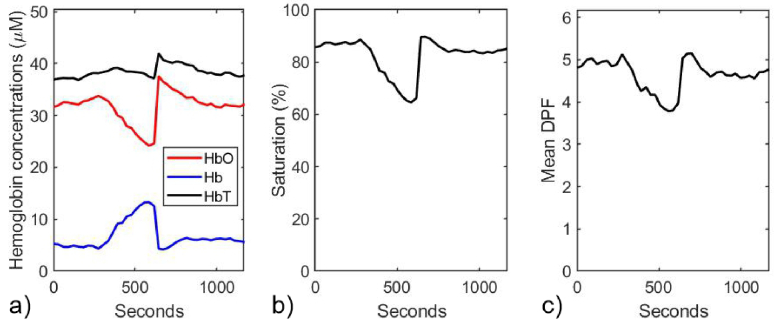
Parameters estimated at 30-second time points during the vascular occlusion on a typical adult volunteer (V1), using a nonlinear least squares fit where the parameters *W*, *L*, *p*, *c_Mel_*, and µ_b_ are held constant at values from a prior fit where *W*, *L*, and *p* are constrained within physiologically-realistic ranges, and where changes in concentrations of HbO_2_ and Hb are constrained relative to time zero so that their sum is equal to the changes in HbT shown in Fig. 11. a) Estimated concentrations of HbO_2_, Hb, and HbT. b) Estimated values of SO_2_. c) Estimated values of the mean DPF.

The fitting process was then repeated a third time, using the additional constraint that the change in the total hemoglobin concentration (relative to that at time zero) is consistent with that exhibited in Fig. 11. The resulting variations in the absolute hemoglobin concentrations, saturation and average DPF are shown in Fig. 12.

If the model results described in section 3.2.4 are considered to be a reliable guide, then the estimates of saturation and wavelength-averaged DPF would be accurate to within around 10%, and the Hb concentration to be accurate to within about 20%. Meanwhile the concentration of HbO_2_ would be likely to be overestimated by as much as 40%.

The results obtained for all five volunteers yielded trends similar to those exhibited in Fig. 12, with the concentrations of both hemoglobins exhibiting broadly consistent ranges of values. These and other derived parameters are summarized in Table 1, and are discussed in the following section.

**Table 1.** Summary of optical parameters derived from forearm vascular occlusion studies on five adult volunteers. The hemoglobin concentrations (c_HbO2_ and c_Hb_), saturation (SO_2_), and DPF show the range of values derived over each entire measurement. Values of water fraction (W), lipid fraction (L), and scattering parameter (p) are constrained within physiologically realistic ranges, while melanin concentration (c_Mel_) and background absorption (µ_b_) are unconstrained.

Volunteer	*c*_HbO2_ (µM)	*c*_Hb_ (µM)	SO_2_ (%)	*W*	*L*	*c_Me_*_l_ (nM)	*p*	DPF	µ_b_ (mm^−1^)
V1	24-37	4-13	64-89	0.52	0.29	22	1.27	3.8-5.1	0.024
V2	51-105	5-34	60-95	0.79	0.15	149	0.71	2.2-3.9	4.5 × 10^−4^
V3	31-69	4-29	52-94	0.78	0.16	201	0.75	2.9-4.1	0.010
V4	70-87	2-8	89-97	0.68	0.29	1	0.71	4.0-4.9	1.4 × 10^−7^
V5	58-67	4-17	77-95	0.60	0.29	1	0.70	4.4-5.6	3.4 × 10^−7^

## 5. Discussion

Previous studies [12] have already shown that measurements of the attenuation spectral gradient *∂A*/*∂λ* is robust to variable and unknown coupling losses which has hitherto limited medical applications of NIRS, and that measurements can be used to estimate absolute concentration of absorbers in homogenous scattering samples. Those concentrations could be recovered with an accuracy of a few percent providing that the DPF was known. In the work described here, the method has been applied for the first time to *in vivo* measurements, where neither the chromophore concentrations nor the DPF are known. In section 2 above, an analysis based on solutions to the time-resolved diffusion equation and a simple power-law model of scattering, yields an approximate expression for *∂A*/*∂λ* which in principle enables chromophore concentrations as well as scattering-dependent parameters (*p* and < *β* >) to be determined simultaneously. An initial validation using the same diffusion model to generate simulated data for a vascular occlusion measurement on muscle demonstrated reasonable recovery of the concentrations and others parameters, although not without constraining some of those to physiological realistic ranges. The concentration in HbO_2_ displayed the greatest error, inevitably due to the fact that the spectral gradient of the specific absorption coefficient of HbO_2_ is relatively featureless and nearly constant over the selected spectral range (see Fig. 2(b)). As already noted previously [15], crosstalk is also expected between the contributions due to melanin and scatter due to their mutual exponential dependence on wavelength. Neither parameter is constrained in the above analysis. The overestimation in HbO_2_ concentration by up to 40% could be reduced using narrower constraints based on prior information, or possibly using a more refined model. Nevertheless, while such error in concentration may seem large when compared with the accuracy achieved when measuring changes in concentration using conventional NIRS, it is a potentially clinically valuable level of accuracy when no other non-invasive method is available to determine absolute concentrations *in vivo*. The apparent ability to estimate the wavelength-averaged DPF to within 10% of the true value is encouraging and perhaps surprising, and potentially offers a novel means of revealing (and imaging) contrast in tissue based on intrinsic variability in DPF.

Validating an expression (i.e. Equation (12)) using the same diffusion model used to derive the expression (albeit while employing different boundary conditions) is suboptimal, and further validation studies are currently in progress using a Monte Carlo model of photon migration in simulated tissues, which is also being used to investigate the influence of noise on measurement accuracy. Nevertheless, although the same diffusion model is used, it should be noted that the equations of Contini *et al* [16]. have, over almost thirty years, been widely validated via comparisons with other models and experimental data.

The decision to use a vascular occlusion measurement on forearm muscle to demonstrate the potential diagnostic potential of the approach was based on the abundance of similar NIRS measurements in the published literature, and the relative simplicity of the experiment. However, the forearm is highly heterogeneous, so determining chromophore concentrations averaged over the sampled volume inevitably yields variable results, even on the same subject when the probe is moved by more than a few millimeters. A small (11 mm) source-detector separation was deliberately chosen to focus the measurement on a specific muscle (the extensor digitorum), as well as to maximize the achievable signal-to-noise ratio.

The results of measurements on five subjects summarized in Table 1 exhibit reasonable consistency in the hemoglobin concentrations, saturation, and DPF. Water fraction, lipid fraction, and scattering power often yield values at one extreme of their constrained range, which suggests that either the constraints are too narrow or (more likely) the fitting process is not able to adequately separate the influences of these parameters. The derived melanin concentration exhibited no obvious correlation with subject skin or hair color or amount of hair on the forearm, although no specific care was taken to avoid placement of the probe over freckles or skin blemishes.

It is very important to note that the values of the three extinction coefficients and two absorption coefficients as a function of wavelength (Fig. 1(a)) extracted from the online database [18] used in this study cannot be considered definitive, and their spectral gradients (Fig. 1(b)), which are key to the success of the approach presented here, are even less well determined. Estimation of the latter depends strongly on the spectral resolution of the measurement of the former, and ideally that resolution should match that of the system used to acquire the spectra of the forearm muscle. Such matching was achieved for previous measurements on absorbing inks in scattering media, which yielded remarkably accurate results [12]. Although existing measurements of specific absorption spectra are sufficient for most NIRS applications, refining the spectral derivative method further may require improved measurements of specific absorption spectra of the chromophores and thus of their spectral gradients.

A number of refinements are possible to the analysis presented here. First, Eq. (12) is derived from a diffusion model which incorporates various approximations. It is possible, in principle, to develop expressions for *∂A*/*∂λ* based on alternative analytical and/or numerical models, which take into account more tissue-like properties and geometries. Second, alternatives to the power-law scattering model could be employed, perhaps derived empirically for specific tissue types. Third, the wavelength-averaged DPF in Eq. (12) could be replaced with a suitable model for the wavelength dependence of the DPF, as investigated by Scholkmann and Wolf [17], although at a cost of additional variables to be determined during the fitting process. And fourth, more prior information could be introduced, such as provided by an independent measurement of the DPF [27,28]. The effect of data noise also needs to be considered in future, as does the potential influence of the chromophore cytochrome which thus far has been ignored.

## 6. Conclusions

This paper presents a means of determining absolute concentrations of chromophores and scattering-related parameters using near-infrared spectroscopy which so far has demonstrated promising results. Further work is required to establish the validity of the approach and the limits of its accuracy when applied to *in vivo* measurements on tissue. A successful method could potentially be applied to a broad range of diagnostic applications, such as identification and assessment of cancer and other abnormal lesions in soft tissue, and differentiating between ischemic and hemorrhagic stroke in the brain.

## Data Availability

Data underlying the results presented in this paper are not publicly available at this time but may be obtained from the author upon reasonable request. The simulation code is also available upon reasonable request.
